# Integrating In Silico and In Vitro Tools for Optimized Antibody Development—Design of Therapeutic Anti-oxMIF Antibodies

**DOI:** 10.3390/antib13040104

**Published:** 2024-12-20

**Authors:** Gregor Rossmueller, Irina Mirkina, Michael Thiele, Alejandro Puchol Tarazona, Florian Rueker, Randolf J. Kerschbaumer, Alexander Schinagl

**Affiliations:** 1 OncoOne Research & Development GmbH, Karl-Farkas-Gasse 22, A-1030 Vienna, Austria; 2 Department of Biotechnology, Institute of Molecular Biotechnology, University of Natural Resources and Life Sciences, Muthgasse 18, A-1190 Vienna, Austria

**Keywords:** antibodies, antibody engineering, antibody optimization, oxMIF, MIF

## Abstract

Background: Rigorous assessment of antibody developability is crucial for optimizing lead candidates before progressing to clinical studies. Recent advances in predictive tools for protein structures, surface properties, stability, and immunogenicity have streamlined the development of new biologics. However, accurate prediction of the impact of single amino acid substitutions on antibody structures remains challenging, due to the diversity of complementarity-determining regions (CDRs), particularly CDR3s. Methods: In this study, we combined in silico tools with in vitro assessments to engineer improved antibodies against the oxidized isoform of the macrophage migration inhibitory factor (oxMIF), building on the first generation anti-oxMIF antibody imalumab. Results: We identified hydrophobic hotspots conferring increased self-interaction and aggregation propensity on imalumab, which unravels its unusually short half-life in humans. By introducing mutations into the variable regions, we addressed these liabilities. Structural prediction tools and molecular dynamics simulations guided the selection of mutations, which were then experimentally validated. The lead candidate antibody, C0083, demonstrated reduced hydrophobicity and self-interaction due to the restructuring of its heavy chain CDR3 loop. Despite these structural changes, C0083 retained target specificity and binding affinity to oxMIF. Conclusions: Altogether, this study shows that a small number of well-selected mutations was sufficient to substantially improve the biophysicochemical properties of imalumab.

## 1. Introduction

Monoclonal antibodies (mAbs) represent a class of biotherapeutics with substantial growth over the last decades, having obtained multiple approvals for clinical use from regulatory agencies like the European Medicines Agency (EMA) and the US Food and Drug Administration (FDA) for the treatment of various human diseases. Notably, they have demonstrated efficacy in treating cancer and autoimmune diseases, through antigen neutralization and the immune-modulating potential of their Fc domains [1]. The advancement of display technologies facilitated the discovery and optimization of antibodies targeting specific antigens [2]. Despite this progress, certain biophysicochemical characteristics, particularly aggregation propensity and hydrophobicity, might often be inferior. Optimizing these parameters can considerably improve mAb manufacturability, including having a positive impact on mAb expression, solubility, stability, half-life, and safety [3,4]. Given the complexity of mAb characteristics, a comprehensive evaluation strategy coupled with alternative methodologies is becoming increasingly important to develop antibody candidates with optimal therapeutic profiles [5]. Minimal amino acids substitutions have often been shown to improve the properties of a protein [6,7,8,9]. The complementarity determining regions (CDRs) of antibodies are responsible for antigen binding. While mAb framework regions are mostly well conserved, CDRs, especially CDR3s, are known for their structural and sequence diversity. Accordingly, these regions can be a substantial source of liabilities. The challenge of predicting biochemical changes caused by single amino acid substitutions in CDRs can be overcome with the help of structure prediction algorithms combined with molecular dynamics (MD) simulation [10,11]. These predictions can thus facilitate mAb development, although they clearly cannot replace experimental validation of the engineered mAbs.

The antibodies described in the present study target the oxidized macrophage migration inhibitory factor (oxMIF), the activated and disease related isoform of the macrophage migration inhibitory factor (MIF) [12]. The ubiquitous nature of MIF and its presence in the circulation of healthy individuals have long been barriers to the development of effective therapies. However, oxidative conditions typical for the inflammatory microenvironment and tumorous tissues were found to convert MIF into its redox-dependent conformational isoform oxMIF. OxMIF is selectively present in the plasma and tissues of patients with inflammatory diseases and in solid tumor lesions, and is increasingly recognized as the isoform responsible for pathological functions of MIF in inflammation and cancer [12,13,14,15,16,17]. MIF to oxMIF conversion exposes hidden epitopes within the compact MIF homo-trimer, enabling specific binding of anti-oxMIF antibodies [15,17].

In preclinical studies, first-generation anti-oxMIF antibodies (BaxB01, BaxG03, BaxM159) neutralized some of the key tumor-promoting activities attributed to MIF in vitro and in vivo [18], and were able to detect oxMIF in primary tumors and metastases of different solid tumors [15]. In a phase I clinical trial (NCT01765790), imalumab (=BAX69, BaxB01) demonstrated an acceptable safety profile, was shown to bind to oxMIF in tumor tissue of metastatic colorectal cancer, and stabilized the disease in 26% of patients [19]. However, this study revealed that imalumab exhibits an unusually short half-life of only 2.3–7.3 days (56–176 h) in humans and showed a non-linear pharmacokinetic (PK) dose relationship, indicating some intrinsic limitations of this first-generation anti-oxMIF antibody [19].

Hydrophobicity and aggregation are considered important attributes in the assessment of antibody developability as hydrophobic patches on the protein surface can catalyze aggregation [20]. High antibody surface hydrophobicity and, consequently, increased aggregation propensity contribute to increased clearance of the therapeutic mAb and have been associated with shorter half-life in vivo [21,22]. Furthermore, they have the potential to elicit severe immunogenic reactions in patients [4,23,24].

Given the promising therapeutic potential of imalumab as an oxMIF-targeting mAb, the objectives of this study were to identify the underlying cause of the short half-life of imalumab, and to develop an optimized second generation therapeutic anti-oxMIF antibody via protein engineering. To this end, we first employed in silico tools, encompassing structure and sequence-based analyses (e.g., hydrophobic hotspot identification, immunogenicity, and posttranslational modification motifs such as oxidation, asparagine deamidation, aspartate isomerization, proteolysis, and N-linked glycosylation), to identify amino acids that may contribute to liabilities of this monoclonal antibody. Subsequently, prediction algorithms and molecular dynamics (MD) simulations were utilized to evaluate the effects of the selected point mutations, derived from structural and sequence analyses, on the characteristics of the antibody. Next, mAb variants carrying the selected mutations were expressed, purified, and evaluated in physicochemical assays. Finally, we crystalized the Fab of the lead candidate C0083 to determine which structural changes account for the improved physicochemical characteristics of this mAb.

## 2. Materials and Methods

### 2.1. Prediction Modeling

The Fv regions of the antibody variants were modeled using the ABodyBuilder [25], available on the SAbPred server from the Oxford Protein Informatics Group (https://opig.stats.ox.ac.uk, accessed on 13 December 2022), which numbers the sequence according to IMGT with ANARCI, chooses the template for the VH and VL domain separately with SAbDab, and predicts the VH-VL orientation using ABangle. The CDR loops are modeled with ABlooper and the side chains are generated with PEARS. The heavy and light chain sequences were trimmed by the algorithm to generate the Fv structure models. The selected framework temples were 3gjf chain H for the heavy chain sequences and 7nx7 chain L for the light chain sequences.

### 2.2. Simulation

The GROMACS [26] software package was used with the GROMOS force-field version 54A7 [27]. The Fab domain of the BaxB01 (=imalumab, BAX69) crystal structure or predicted ABodyBuilder models for the novel mAbs were solvated in a cubic water box using the SPC water model. The protonation state of the amino acids was chosen to resemble a neutral pH: Lys and Arg protonated and Asp and Glu deprotonated. For His, the possible protonation of ND1, NE2, or both was based on the optimal hydrogen bond conformation (maximum donor–acceptor distance of 0.3 nm and minimum donor–acceptor angle of 135°). Subsequently the protein charges were neutralized by adding counter ions, and after, 150 mM NaCl was added. Unless otherwise stated, the used cut-off scheme was Verlet and Particle Mash Ewald for long-range electrostatic interactions. The short-range electrostatic and van der Waals cut-offs were set to 1.4 nm.

The structures were energy minimized using the Steepest Descent algorithm until the maximum force was below 500 kJ/mol/nm. The initial velocities for the next step were randomly selected from a Maxwell–Boltzmann distribution at 300 K. The positions of heavy atoms were restrained during equilibration by 1000 kJ/mol nm^2^. Equilibration of the system was performed for 100 ps applying an NVT ensemble at 300 K using the V-Rescale thermostat [28] with relaxation times of τ_T_ = 0.1 ps and for 100 ps applying an NPT ensemble using the Parrinello–Rahman barostat [29] at 1 bar with τ_P_ = 2 ps. The production simulation run was performed for 10 ns using Nose–Hoover thermostat and Parrinello–Rahman barostat with relaxation times of 0.1 and 5 ps, respectively. The long-range electrostatics were computed using the smooth Particle-Mesh Ewald (SPME) method. A 1.4 nm cutoff scheme was used. Translational motion of the center of mass of the protein was removed over 100 steps. We compared the results of 10, 30, and 50 ns simulation runs and found no significant differences between the calculated hydrophobicity values for the amino acids. The RMSD of all structures to the initial structure within each simulation run was calculated in GROMACS as a quality control to ensure the structures converged. To calculate the surface hydrophobicity, a representative structure of the cluster with the most structures assigned was selected using the GROMACS cluster function with the gromos [30] method.

### 2.3. Surface Hydrophobicity Calculation

The hydrophobicity prediction was carried out by calculating the solvent accessible area of the residues and multiplying them by the specific residue hydrophobicity according to the Black and Mould [31] hydrophobicity scale. All computations were executed utilizing the Python programming language and API of PyMOL Molecular Graphics System (version 2.5.4, Schrödinger, LLC, New York, NY, USA).

### 2.4. Immunogenicity Prediction

The MHCII binding predictions were made using the IEDB analysis resource Consensus tool [32,33]. A preselected group of the 8 most frequent DR alleles was chosen: HLA-DRB1*01:01, HLA-DRB1*03:01, HLA-DRB1*04:01, HLA-DRB1*07:01, HLA-DRB1*08:02, HLA-DRB1*11:01, HLA-DRB1*13:02, and HLA-DRB1*15:01 [33].

### 2.5. MIF and mAb Expression and Purification

These methods were performed according to Rossmueller and Mirkina et al. [14].

Briefly, for MIF expression and purification, Shuffle T7 express lysY competent *E. coli* (New England Biolabs, Ipswich, MA, USA) were transfected with a Champion pET303/CT plasmid (Thermo Fisher Scientific, Waltham, MA, USA) containing the human MIF cDNA. The sequence was taken from https://uniprot.org (accessed 21 January 2019) under accession code P14174. LB medium (Thermo Fisher Scientific) was inoculated with transfected *E. coli* and cultivated o/n at 37 °C. Then, an aliquot was transferred to Super Optimal Broth (VWR, Radnor, PA, USA), which was supplemented with 10 mM MgSO_4_, 10 mM MgCl_2_, and 2.5 mM KCl, and adjusted to pH 7.0. After o/n incubation at 30 °C the cells were harvested by centrifugation and lysed. The cleared lysate was applied to 5 mL HiTrap DEAE FF (Cytiva, Marlborough, MA, USA) columns to remove the host cell proteins and the flow-through was applied to 5 mL HiTrap SP FF (Cytiva) columns to polish the MIF fraction. The MIF sample was then buffer exchanged to 1× PBS (Thermo Fisher Scientific) and stored at 4 °C.

The DNAs encoding heavy and light chains for all the mAb variants and for the Fab were de novo gene-synthesized at GeneArt/Thermo Fisher Scientific and subcloned into the pcDNA 3.4 TOPO mammalian expression vector (Thermo Fisher Scientific). ExpiCHO-S cells (Thermo Fisher Scientific) were transfected with the plasmids according to the max titer protocol of the manufacturer and the supernatants were harvested 13–14 days post transfection. The antibodies were purified from supernatants by using MabSelect Prism A columns (Cytiva), buffer-exchanged to PBS, and sterile filtered prior to storage at −20 °C.

### 2.6. Anti-oxMIF mAb ELISA

This ELISA was performed as described previously [14,34]. In short, human MIF-coated microtiter plates (immobilization of MIF disrupts the compact trimeric structure making the epitope for the anti-oxMIF mAbs accessible [17]) were incubated with a serial dilution of antibodies and bound antibodies were detected with an anti-human Fc IgG HRP conjugate (Thermo Fisher Scientific, Waltham, MA, USA) and tetramethylbenzidine (Thermo Fisher Scientific) as substrate. The reaction was stopped with 3 M sulfuric acid and absorbance was measured at 450 and 650 nm on an Infinite PRO 200 plate reader (Tecan, Männedorf, ZH, Switzerland). For data analysis, the absorbance values at 650 nm were subtracted from 450 nm, and the concentration vs. signal data were fitted by a 4-parameter logistic function using GraphPad Prism (version 10.2; GraphPad Software, Bosten, MA, USA). The EC_50_ value of the parental antibody (C0008, imalumab) was set to 100% potency. The potency was calculated by potency% = log(EC_50_, C0008)/log(EC_50_, sample) × 100.

### 2.7. Differential oxMIF-Binding ELISA

The oxMIF specificity was assessed in accordance with Schinagl et al. [35], with the following adjustments indicated by Rossmueller and Mirkina et al. [14]: antibodies were immobilized at a concentration of 15 nM in 100 µL per well, and 50 or 100 ng/mL of TNB-MIF was used as a surrogate for oxMIF.

### 2.8. Size-Exclusion Chromatography

The purity of the antibodies was assessed using an ENrich SEC 650, 10 × 300 mm gel-filtration column (Bio-Rad, Hercules, CA, USA) on an NGC Quest 10 chromatography system (Bio-Rad). The samples were diluted to a final concentration of 0.5 mg/mL in 1× PBS. One hundred µL samples were injected and the column flow rate was maintained at 1.5 mL/min at RT. Protein peaks were monitored using absorbance at 280 nm and the spectra were analyzed by the ChromLab Software (version 6.0; Bio-Rad).

### 2.9. Hydrophobic Interaction Chromatography

All samples were diluted to a final concentration of 1 mg/mL antibody, 750 mM ammonium sulfate and pH 6.9 with a 50 mM sodium phosphate, 1.5 M ammonium sulfate pH 6.9 buffer. One hundred µL of sample were injected onto a 1 mL HiTrap Butyl HP column (Cytiva, Marlborough, MA, USA). All equilibration steps were carried out with a 50 mM phosphate, 1125 mM ammonium sulfate pH 7.0 buffer. The elution was performed by a linear gradient over 20 CV to 50 mM phosphate, 20% isopropanol, pH 7.0. The protein peaks were monitored at 280 nm. The runs were performed on an NGC Quest 10 chromatography system (Bio-Rad, Hercules, CA, USA) and the chromatograms were analyzed by ChromLab Software (version 6.0; Bio-Rad).

### 2.10. AC-SINS

The method was adapted from Liu et al. [36] The capture antibody, goat anti-human- IgG Fc-specific (Thermo Fisher Scientific, Waltham, MA, USA), was buffer exchanged to coating buffer 20 mM NaAc/Ac at pH 4.3, using Zeba Spin columns (Thermo Fisher Scientific) as per the manufacturer’s guidelines. Post-buffer exchange, the capture antibody was diluted to a concentration of 0.4 mg/mL with the same coating buffer. Next, the capture antibody was combined with gold nanoparticles (GNPs, Merck, Darmstadt, HE, Germany) of 20 nm diameter at OD = 1 at a ratio of 1:12.25 and allowed to incubate for 1 h at room temperature. A stock solution of 20 mg/mL thiolated PEG 2000 (Merck) was prepared in coating buffer. This stock solution was added to the GNPs, at a final concentration of 1 µM. The reaction was left to incubate for 2 h at room temperature, and the GNPs were subsequently stored at 4 °C until further use. For concentrating the anti-human GNPs, a 13 mm diameter PVDF filter with a 0.2 µm pore size was used. The GNPs were retained on the membrane due to electrostatic interactions and eluted from the filter by aspirating with 1/10 of the original volume of PBS (10 × anti-human GNPs). The antibody samples were diluted to a concentration of 100 µg/mL with PBS, and 40 µL of this solution were incubated with 10 µL of 10 × anti-human GNPs for 2 h in a 384-well plate (Geiner Bio-One, Kremsmünster, OOE, Austria). Subsequently, the absorbance within the range of 480 to 580 nm was measured with 2 nm intervals and 10 flashes per measurement on an Infinite 200 PRO plate reader (Tecan, Männedorf, ZH, Switzerland).

The absorbance data were fitted using a second-order polynomial curve in GraphPad Prism (version 10.2; GraphPad Software, Bosten, MA, USA) within the range of 500 to 560 nm (the quadratic range). The first derivative of the curve was then calculated (y = ax^2^ + bx + c; y’ = 2ax + b), and the x-value at which y = 0 (x = (0 − b)/2a) represented the maximum of the quadratic curve. This value was compared to the negative control beats and the shift in absorbance maximum was reported.

### 2.11. X-Ray Crystallography

Purified C0083 Fab in PBS was concentrated to 55 mg/mL using Amicon Ultra centrifugal filter units (Merck, Darmstadt, HE, Germany). Pre-crystallization tests were performed at 27.9 mg/mL in PEG (0.1 M Tris-HCl, pH 8.5, 0.2 M MgCl_2_, 30% *w/v* PEG4000) and NH_4_Cl (0.1 M Tris-HCl, pH 8.5, 2 M NH_4_Cl) and monitored for aggregation. Crystallization was carried out using the sitting drop vapor diffusion method, utilizing Intelli-Plate 96-3 LVR plates (Hampton Research, Aliso Viejo, CA, USA). The crystallization setup, employing commercially available screens, was automated using the mosquito crystallization robot (STP LabTech, Melbourn, CAM, UK). The reservoir was filled with 40 µL of precipitant solution and different ratios of Fab at 9.9 mg/mL to precipitant (150:200 nL, 200:200 nL and 250:200 nL) were applied. Crystallization plates were stored in a Formulatrix RI-1000 imaging device (FORMULATRIX, Oberursel, HE, Germany) at 20 °C. Promising conditions (0.1 M Bis-Tris, pH 6.5, 0.2 M MgCl_2_, 25% (*w/v*) PEG3350 and 0.1 M sodium acetate, pH 5, 0.2 M CaCl_2_, 20% (*w/v*) PEG6000) were optimized by adjusting the pH by ±0.5 and the precipitant concentration by ±5%. Crystals were soaked in a mother liquor solution supplemented with 20% 2-methyl-2,4-pentanediol (MPD), harvested using cryo-loops, and rapidly frozen using liquid nitrogen.

The datasets were collected at beamline ID30B [37] of the European Synchrotron Radiation Facility (ESRF) in Grenoble, France, and were collected at 100 K using a EIGER2 X 9 M 450 µm Si sensor (Dectris, Baden, AG, Switzerland). The data DOI is 10.15151/ESRF-ES-1163229127. The highest-quality dataset was obtained from a crystal in 0.1 M Bis-Tris, pH 6.33, 25.545% (*w/v*) PEG 3350, 0.2 M MgCl_2_. The dataset was processed with XDS [38]. The phase problem was solved by molecular replacement employing phenix.phaser [39] utilizing the search model 6FOE (BaxB01). Further refinement was carried out through iterative cycles of manual model building using COOT [40] and maximum likelihood refinement using phenix.refine [39]. Phenix.refine converted intensities into amplitudes using the French and Wilson algorithm [41]. The determination of the final high-resolution cutoff was based on performing paired refinement utilizing the PDB_REDO webserver [42]. The last stages of refinement encompassed Translation Liberation Screw (TLS) parameters, isotropic B-factor model, automated addition of hydrogens and water molecules, optimization of X-ray/ADP weight, and optimization of X-ray/stereochemistry weight. Figures were prepared with PyMOL Molecular Graphics System (Version 2.5.4, Schrödinger, LLC). The structural data from this study has been submitted to the RCSB Protein Data Bank (PDB) and can be accessed with the accession number 9FQO at http://rcsb.org (accessed on 22 November 2019).

The normalized b-factors of the C0083 and imalumab crystal structures were calculated by following formula: normalized b-factor = (b-factor − μ)/σ, according to Johnson and Gallego et al. [43], where µ and σ are the mean and standard deviation of the b-factors, respectively.

### 2.12. Mass Spectrometry

One hundred µg of sample were mixed with 8 M guanidine hydrochloride (Sigma Aldrich, St. Louis, MO, USA) to a final concentration of 6 M. To reduce the disulfide bonds, 1 M dithiothreitol (Sigma Aldrich, St. Louis, MO, USA) to a final concentration of 20 mM was added. The sample was incubated at 56 °C for 30 min. The cysteines were alkylated with 50 mM iodoacetamide (Sigma Aldrich, St. Louis, MO, USA) for 45 min at room temperature. Prior to digestion, the sample was buffer-exchanged to 50 mM Tris-HCl pH 7.0. The digestion enzyme Trypsin (Aladdin Scientific, Riverside, CA, USA) was added in a ration of 1:25 (*w*:*w*), mixed and incubated at 37 °C for 16 h. The digested sample was separated by a reversed-phase high performance liquid chromatograph C18 column (Waters, Milford, MA, USA) with a 0.1% formic acid (Fisher Scientific, Schwerte, NW, Germany) in water to 0.1% formic acid in acetonitrile (Fisher Scientific) gradient, and then subjected to mass spectrometry analysis on a Q Exactive mass spectrometer (Thermo Fisher Scientific, Waltham, MA, USA). Peptide identifications were performed by searching the processed data against this project sequence using BioPharma Finder software (version 5.1; Thermo Fisher Scientific). By comparing the mass-to-charge ratio (*m/z*) of the primary ions and the secondary fragment ions with the theoretical value of the peptide, the sites and types of post-translational modifications were determined. The modification ratio was calculated from the area of the extraction ion chromatogram of the corresponding peptide.

### 2.13. Writing Assistance Software

Grammatical and semantic correctness were checked using the DeepL Write module (version 24.11.4.14424).

## 3. Results

### 3.1. In Silico Analysis and Sequence-Optimization to Generate Improved Second Generation Anti-oxMIF mAbs

The variable heavy (VH) and light (VL) chain sequences of the first-generation anti-oxMIF mAb, imalumab (=BAX69, BaxB01), were screened in silico for potential sequence liabilities. In addition, the crystal structure of BaxB01 Fab (PDB ID: 6FOE) was analyzed for structural liabilities as light chain (LC) and heavy chain (HC) sequences of BaxB01 are identical to the published imalumab Fab sequence (BAX69, GenBank JB325049.1 (LC), GenBank JB325055.1 (HC)). Notably, the Fab fragment of imalumab crystalized as Fab dimers, whereas the dimers are stabilized by hydrophobic interactions.

### 3.2. Hydrophobicity and Aggregation Potential

**Figure 1:**
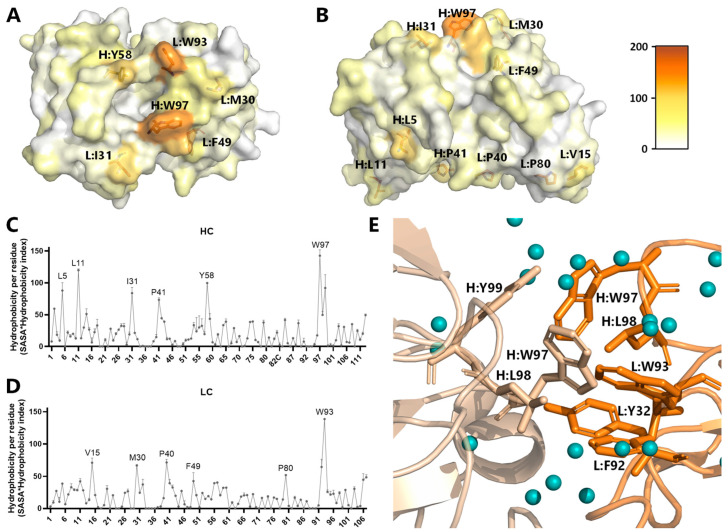
**In silico analysis of imalumab crystal structure.** (**A**) Top view of the imalumab Fv region in VH/VL orientation from crystal structure (PDB:6FOE, chains A and B) and (**B**) side view of the Fv region in VH/VL orientation. The amino acids are colored according to the calculated hydrophobicity index (hydrophilic/white to hydrophobic/orange) using the Black and Mould hydrophobicity scale and the solvent accessible area to calculate the hydrophobicity of the individual amino acids. (**C**,**D**) The calculated hydrophobicity per residue reported as mean value ± SD from the 6FOE crystal structure chains A and H (HC) and chains B and L (LC). The identified hotspots are marked in the graph of the HC (**C**) and LC (**D**). (**E**) The crystal structure 6FOE demonstrated that imalumab Fabs crystallized as dimers (beige/orange) facilitated by hydrophobic interactions. The water molecules around the hydrophobic interaction site are shown as blue spheres. The interaction of the two Fabs is driven by H:L98, H:W97, H:Y99, L:Y32, L:F92, and L:W93. (**A**,**B**,**E**) H—heavy chain; L—light chain.

Referring to the reported correlation between increased clearance and hydrophobicity of an antibody [21,22], we first screened the structure of imalumab for hydrophobic hotspots. For this, we calculated the solvent accessible surface area (SASA) per residue in PyMOL (version 2.5.4) [44] and multiplied it by the hydrophobicity index of the respective amino acid derived from the Black and Mould scale [31,45] (Figure 1A–D). The analysis revealed hydrophobic hotspots and patches within the variable domains of both the HC and LC. Specifically, residues H:L11, H:W97, and L:W93 exhibited elevated hydrophobicity scores, while residues H:L5, H:I31, H:P41, H:Y58, L:V15, L:M30, L:P40, L:F49, and L:P80 displayed moderate hydrophobicity scores. Consequently, these amino acids were considered as targets for engineering.

**Figure 2:**
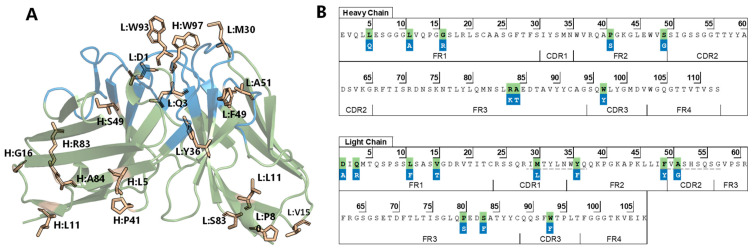
**Crystal structure and sequence of imalumab variable domains.** (**A**) Crystal structure (6FOE) of BaxB01 (=imalumab) presented as cartoon (green) with CDRs (Kabat) in blue, highlighting amino acids selected for mutation as sticks (beige). (**B**) The VH and VL sequences of imalumab with annotated CDRs and framework (FR) regions according to Kabat. The selected amino acids for mutation are marked in green with the respective amino acid substitutions highlighted in blue below. Predicted T-cell epitopes are underlined with gray dashed lines in the LC.

Some of these amino acids (HC: W97, L98, H99; LC: Y32, F92, W93, L98) are involved in the formation of a hydrophobic pocket in the solved crystal structure of the BaxB01 Fab, showing two interacting Fabs in the asymmetric unit (Figure 1E). Therefore, we supposed that the central amino acids involved in this hydrophobic interaction, H:W97 and L:W93, might be the main contributors to the hydrophobicity of imalumab. Interestingly, both amino acids, H:W97 and L:W93, reside within the CDR3 (Figure 2) of the respective chains. The CDR3s are regarded as being the most important for determining the antigen specificity, and, thus, mutations in these regions should be performed with caution as they can substantially disrupt the functional antibody binding [46].

### 3.3. Post-Translational Modifications

Tryptophan and methionine are commonly recognized as amino acids prone to oxidation, which can pose a threat to the stability of mAbs and ultimately compromise their efficacy and safety [47]. Residues H:W97, L:M30 and L:W93 are highly solvent-exposed (Figure 2A) making them susceptible to oxidation and were, thus, selected as candidates for mutations. Sequence analysis for asparagine deamidation involved screening for motifs SNG, ENN, LNG, LNN as reported by Chelius et al. [48]. Additionally, N-linked glycosylation motifs (Asn-Xaa-Ser/Thr, excluding Pro at position Xaa) were examined using NetGlyc 1.0 [49]. Further, sequences were screened for proteolysis motif DP, and aspartate isomerization motifs DS, DN, DQ, DK, DL, as described in Vlasak et al. [50]. However, besides the above-reported oxidation-prone residues, the sequence analysis did not reveal any significant sites of risk.

### 3.4. In Silico Immunogenicity Risk Assessment 

Presentation of peptide sequences in the groove of MHC Class II molecules leads to the activation of CD4+ T-cells and an immunogenic response. In order to reduce this response, therapeutic proteins can be designed to avoid the incorporation of such potential T-cell-activating epitopes by reducing the affinity of binding to the MHC Class II molecules. The VH and VL sequences of imalumab were therefore screened for high affinity (IC_50_ < 50 nM) MHC II T-cell binding peptides using TepiTool [51]. The respective closest germline sequences IGHV3-23*01 and IGKV1-39*01, as identified by using IgBlast (https://www.ncbi.nlm.nih.gov/igblast/, accessed on 22 November 2019), were analyzed for comparison, as human germlines are unlikely to be immunogenic. Notably, the heavy chain did not show any high affinity MHC class II epitopes. In the LC we identified two potential core epitopes: (1) I_29_MTYLNWYQ_37_ (HLA-DRB1*15:01) and (2) F_49_VASHSQSG_57_ (HLA-DRB1*01:01 and HLA-DRB1*15:01) although the first is not fully exposed (Figure 2B).

### 3.5. Framework Optimization

By comparing the imalumab VL and VH domains to all available human antibodies in AbYsis [52] and to VH3 and Vκ1 sequences in the IMGT germline repository [53], we searched for rare amino acids within the frameworks of imalumab. The following amino acids were identified to be uncommon in the germlines or to have hydrophobic characteristics: P41, S49, R83, and A84 of the VH, and D1, Q3, L11, V15, P80, and S83 of the VL (Figure 2).

### 3.6. Design of Optimized mAb Variants

Analysis of imalumab VH and VL sequences for hydrophobic and aggregation-prone hotspots identified the following high risk amino acids: H:L5, H:W97, L:M30, L:F49, L:P80, and L:W93. To address the revealed liabilities, we replaced H:W97 with tyrosine (H:W97Y) and L:W93 with phenylalanine (L:W93F) to maintain aromatic amino acids at these positions, as those might be essential for oxMIF specificity and affinity. The relative hydrophobicity of phenylalanine compared to tryptophan varies based on the hydrophobicity scale used. However, in many modern and widely accepted scales (Kyte & Doolittle [54], Eisenberg [55], Black & Mould [31]), phenylalanine is regarded as being more hydrophobic [56]. Consequently, the impact of this substitution on hydrophobicity needs experimental validation. The crystal structure revealed that amino acids H:W97 and L:W93 are involved in the interaction of two imalumab Fabs, leading to the formation of a hydrophobic pocket. Therefore, substituting H:W97 with tyrosine (Y) to introduce a polar hydroxyl group at this position is expected to reduce the surface hydrophobicity, thus decreasing the propensity for self-interaction. In addition to forming a hydrophobicity and aggregation hotspot, L:W93 and H:W97 were also found to be prone to oxidation. Accordingly, the introduced mutations L:W93F and H:W97Y should also minimize the oxidation risk. Mutations H:L5Q, L:M30L, L:F49Y, and L:P80S were introduced based on the following rational: high prevalence of the more hydrophilic glutamine at position 5 (Q5) and tyrosine at position 49 (Y49) in the germline repertoire of the VH and VL, respectively, according to the IMGT database [53], reduced oxidation propensity of leucine (L30) compared to the oxidation-prone methionine (M30), and higher hydrophilicity of serine (S80) compared to proline (P80).

Additional amino acids identified as potential candidates for reducing surface hydrophobicity were L11, I31, P41, and Y58 of the HC, as well as V15 and P40 of the LC. However, all these amino acids were not modified in our initial screen due to either their high conservation, or because they are not part of the identified aggregation hot spot (Figure 1E).

Finally, we sought to remove the predicted high affinity T-cell epitopes. Mutation L:Y36F led to the removal of predicted T-cell epitope 1, whereas mutation L:A51G enabled the removal of predicted T-cell epitope 2.

In summary, our analysis of variable regions of imalumab revealed hotspots associated with elevated surface hydrophobicity and a propensity for aggregation, high affinity T-cell epitopes, oxidation sites, and several uncommon amino acid residues in the frameworks (Figure 2A,B). We therefore designed mAb variants carrying the above selected mutations (Table 1) and screened them using in silico modeling tools.

### 3.7. In Silico Modeling of Optimized mAb Variants

**Figure 3:**
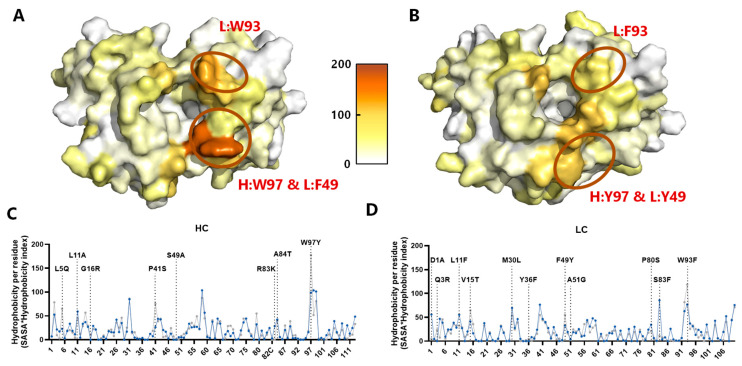
**In silico models of the parental anti-oxMIF antibody and engineered variant Fv-M1**. (**A**) One Fab, extracted (chains A + B) from the BaxB01 (6FOE, imalumab) crystal structure, and (**B**) an Fv (Fv-M1) containing all proposed mutations modeled by ABodyBuilder were simulated in PBS like conditions for 10 ns (One representative structure from two independent simulations is shown). Top view of the structure of the (**A**) imalumab Fv region and (**B**) Fv-M1 in VH/VL orientation with the amino acids colored according to the calculated hydrophobicity index (hydrophilic/white to hydrophobic/orange) using the Black and Mould hydrophobicity scale and the solvent accessible area to calculate the hydrophobicity of the individual amino acids is shown. (**C**,**D**) The calculated hydrophobicity per residue in the VH (**C**) and VL (**D**) reported as mean value ± SD from imalumab (6FOE, chains A and H (VH) and chains B and L (VL), gray lines) and Fv-M1 (blue lines) were plotted against the amino acid number according to Kabat.

The Fab region of imalumab (extracted from the crystal structure of BaxB01, 6FOE) and the Fvs containing the proposed mutations according to Figure 2 were simulated for 10 ns (Figure 3A) using the structure prediction models of AbodyBuilder [25], and compared with each other. The model of Fv-M1 harboring all the suggested mutations aiming to optimize the antibody (HC: L5Q, L11A, G16R, P41S, S49G, R83K, A84T, W97Y; LC: D1A, Q3R, L11F, V15T, M30L, Y36F, F49Y, A51G, P80S, S83F, W93F) is shown as a representative (Figure 3B). The model exhibited minimal variation in residue positions to the parental antibody imalumab with a root mean square deviation (RMSD) (atom-to-atom alignment) of 1.49 Å, while the model of imalumab was highly similar to its own solved crystal structure (RMSD = 0.60 Å). However, the beneficial effects of the introduced mutations were evident, as L5Q, P41S, and W97Y in the heavy chain (HC) and V15T, F49Y, and W93F in the light chain (LC) exhibited a reduced hydrophobicity index compared to imalumab (Figure 3C,D). Given that the most hydrophobic amino acids, H:W97 and L:W93, substantially contribute to the surface hydrophobicity of imalumab, the introduced mutations—H:W97Y and L:W93F—were expected to reduce the overall hydrophobicity. However, the total hydrophobicity scores, defined as the sum of the hydrophobicity scores of all individual residues, were similar for the VH (2292 vs. 2385), whereas for the VL it was even higher (2159 vs. 1879) when comparing Fv-M1 to imalumab, respectively. Additional Fv models generated for the mAb variants listed in Table 1 yielded very similar results to those of model Fv-M1. In light of these contradictory results, the in vitro physicochemical characterization of mAb variants seemed to be indispensable to evaluate the effect of the selected mutations.

### 3.8. Physicochemical Characterization of Sequence-Optimized mAb Variants

To validate the predictions made by computational modeling, the above-described VH and VL point mutations were combined in two screening rounds resulting in a total of 25 antibody variants (Table 1), which were expressed in ExpiCHO-S cells, purified using protein A chromatography, and tested in a set of biophysicochemical assays in a head-to- head comparison with in-house produced imalumab lacking the C-terminal lysine (termed C0008).

**Figure 4:**
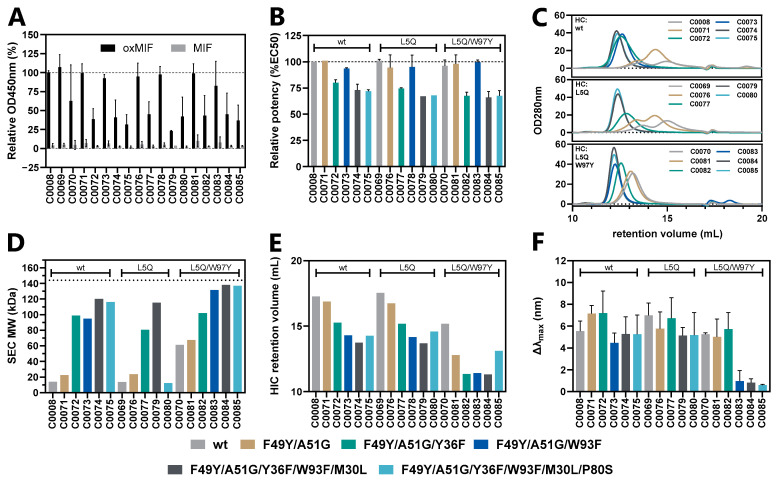
**Physicochemical characterization of the 1st screen of anti-oxMIF mAb variants.** (**A**) The specificity of the mAbs for oxMIF was tested in the differential oxMIF binding ELISA. In this assay, mAbs were immobilized on a microtiter plate, and the binding of oxMIF (black bars) in solution was compared to the binding of MIF (gray bars). Absorbance values were normalized to C0008 (=100%). (**B**) The apparent affinity of the mAbs against oxMIF was determined in anti-oxMIF mAb ELISA using human (ox)MIF immobilized on a microtiter plate. The EC_50_ values from the 4-parameter logistic fit curve were normalized to C0008 (=100%) and reported as relative potency. (**C**) The purity of the samples was assessed by SEC, and the molecular weight (MW) (**D**) of the antibodies was determined through peak integration and comparison to a calibration standard. (**E**) The hydrophobicity was determined by HIC, and the self-interaction propensity (**F**) was assessed by AC-SINS using anti-human IgG coated gold nanoparticles (GNPs). The peak maxima of the spectrum were compared to coated GNPs alone. The color codes according to the LC mutations of the antibody variants are shown at the bottom of the figure, and the antibodies are grouped according to the HC mutations indicated above the bars (**B**–**F**). (**A**,**B**,**F**) Mean ± SD values of *n* ≥ 2 independent experiments are shown. (**C**–**E**) One representative from 2 independent experiments is shown.

In an initial (first) screening, we tested mAb variants C0069-C0085. These variants carry mutations predicted to reduce hydrophobicity, oxidation risk, and to eliminate high affinity T-cell epitopes. First, we investigated whether the variants retained their specificity and affinity to oxMIF by two ELISA methods. We found that all variants retained specificity for oxMIF over MIF by a differential oxMIF-binding ELISA (Figure 4A). However, variants carrying mutation L:Y36F within L:FRW2 (C0072, C0074, C0075, C0077, C0079, C0080, C0082, C0084, and C0085, Table 1) showed reduced binding to oxMIF compared to C0008 and other variants (Table 1) lacking this mutation. In another ELISA method we determined the apparent affinity of the mAb variants in solution towards plate-immobilized human (ox)MIF. During immobilization of MIF, the compact trimeric structure becomes disrupted, making the epitope for the anti-oxMIF mAbs accessible [17]. This assay confirmed a substantially reduced apparent affinity (EC_50_ 518–1609 pM) of all variants containing mutation L:Y36F, compared to C0008 and to variants without this mutation (EC_50_ 148–260 pM) (Figure 4B).

Determination of melting temperatures of mAb variants in nanoDSF (Differential Scanning Fluorimetry), a technique to assess protein stability, revealed a negative impact of the L:Y36F mutation: all variants harboring this mutation exhibited reduced thermal stability (Figure S1) of their Fab domain in comparison to C0008. These data indicate that the position L:Y36 is crucial for proper light chain assembly.

Next, we assessed whether the selected point mutations reduced mAb hydrophobicity and aggregation potential. The SDS-PAGE (Figure S2) confirmed high mAb purity and 1:1 heavy and light chain pairing. However, size exclusion chromatography (SEC) analysis (Figure 4C,D) showed that the parental antibody (C0008) and most variants eluted at volumes inconsistent with the molecular weight of IgGs. This suggests that hydrophobic interactions between the mAbs and the stationary phase (resin) occurred, as PBS was used as a mobile phase to avoid ionic interactions.

C0008 showed a retention volume near the column volume of the SEC column (~15 mL, Figure 4C,D), indicating unspecific interactions with the column resin that lead to a delayed elution. In addition, aggregation was observed by the presence of an additional peak at ~13.5 mL. The introduction of mutations in the VL, specifically L:F49Y and L:A51G (C0071), led to a decrease in the retention volume relative to the parental antibody (−0.6 mL). Further, adding mutations L:Y36F or L:W93F in combination with L:F49Y and A51G (C0072 and C0073, respectively) resulted in an additional decrease in retention volume (−2.4 and −2.3 mL, respectively). The mutation H:L5Q (C0069) did not substantially change the retentional volume in comparison to the parental antibody (+0.1 mL), while the combination of H:L5Q and H:W97Y (C0070) led to a decreased retention volume (−1.8 mL). The greatest effect was achieved by combining L:W93F and H:W97Y in C0083, C0084 and C0085 (−2.7, −2.8 and −2.8 mL, respectively). These variants demonstrated retention volumes close to the theoretical molecular weight of a human IgG1 (~144 kDa, Figure 4D), as verified against a molecular weight standard. In addition, antibody dimers and aggregates were markedly reduced in these samples, evidenced by a single symmetric peak, whereas other variants displayed at least one additional peak at a higher molecular weight (Figure 4C). These data suggest that the selected mutations substantially reduced mAb surface hydrophobicity.

To validate these results, we analyzed the mAb variants by hydrophobic interaction chromatography (HIC), a more specific and sensitive method to determine differences in the hydrophobicity of biomolecules (Figure 4E). HIC provided a more detailed understanding of the hydrophobicity-reducing effects of individual mutations and their combinations, as reduced retention volumes on the HIC column indicate reduced hydrophobicity. Specifically, mutation L:F49Y in combination with L:A51G decreased the retention volume by 0.4 mL (C0071) and further up to 4.5 mL when combined with H:L5Q and H:W97Y (C0081). Mab variants containing mutations H:L5Q/W97Y/L:F49Y/A51G in combination with L:Y36F (C0082), with L:W93F (C0083), or with L:Y36F/W93F/M30L (C0084), demonstrated the greatest reduction in surface hydrophobicity. However, the mutation L:P80S, which introduced a polar hydroxy group, resulted in a minor elevation of the hydrophobicity of C0085 compared to C0084 (Figure 4E). HIC analysis confirmed the correlation between lower retention volumes during SEC and reduced hydrophobicity.

Finally, we evaluated the tendency of the anti-oxMIF mAb variants to aggregate using affinity-capture self-interaction nanoparticle spectroscopy (AC-SINS). This method quantifies the self-interaction of proteins as a measure of their aggregation potential. As depicted in Figure 4F, C0083, C0084, and C0085 (Δλ_max_ 0.97, 0.83, and 0.61 nm, respectively) demonstrated the lowest tendency for self-interaction, illustrating the importance of both CDR3 mutations, L:W93F and H:W97Y, for the reduction in self-interaction, and thus the aggregation propensity of the mAb variants.

**Figure 5:**
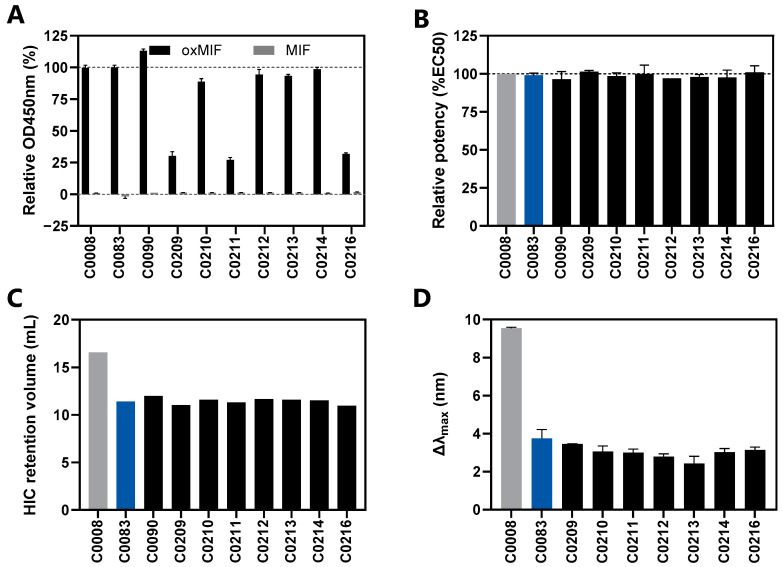
**Physicochemical characterization of the 2nd screen mAb variants in comparison to C0008 and C0083.** (**A**) The specificity of mAb variants against oxMIF was determined by the differential binding ELISA. We compared the binding of oxMIF (black bars) to the binding of neutral MIF (gray bars) in solution with the antibodies immobilized onto the plate. The absorbance values were normalized to the oxMIF binding signal of C0008 (=imalumab). (**B**) The potency of mAb variants was determined in ELISA with immobilized human (ox)MIF and mAbs applied in solution. The experimental data were fitted with a four-parameter logistic function and the corresponding EC_50_ values were normalized to the EC_50_ of C0008 (=100%). (**C**) The surface hydrophobicity was determined by HIC and the self-interaction propensity (**D**) by AC-SINS. (**A**,**B**,**D**) Mean ± SD values of *n* ≥ 2 independent experiments are shown. (**C**) One representative from 2 independent experiments is shown.

In the follow-up (second) screening round, additional mAb variants were generated based on the results obtained in the initial (first) screening. Variant C0090 incorporates all previously tested mutations excluding L:Y36F, which showed a deleterious effect on antigen binding and mAb stability. Variants C0209-C0214 and C0216 include at least the two CDR3 mutations (H:W97Y; L:W93F), which were found to be particularly efficient in reducing hydrophobicity and aggregation potential. Furthermore, individual amino acids in the framework regions, identified as being uncommon in the germlines (VH3 and VK1 families in IMGT [53] and human antibody repertoire in AbYsis [52]) or to exhibit hydrophobic characteristics, were replaced by the most abundant and less hydrophobic amino acids from the closest germlines. Specifically, the substitutions were as follows: VH: L11A, G16R, P41S, S49G/A, R83K and A84T; VL: D1A, Q3R, L11F, V15T and S83F. All of these variants (Table 1) were then assessed in the same biophysiochemical assays as described for the initial screen (Figure 5).

The absence of mutation L:Y36F in the variant C0090 resulted in preserved oxMIF-binding, yielding EC_50_ values similar to the parental antibody (Figure 5A,B). Interestingly, no improvement in hydrophobicity over C0085 or C0083 was observed (Figure 5C) and none of the combinations of additional mutations introduced into the framework regions led to a significant improvement in hydrophobicity or aggregation potential (Figure 5C,D). Instead, three candidates (C0209, C0211, C0216) showed a lower signal in the differential oxMIF ELISA (Figure 5A), whereas the anti-oxMIF binding ELISA showed no difference across all variants (Figure 5B).

Since L:M30 was predicted to be susceptible to oxidation potentially affecting the antibody stability, we assessed its oxidation experimentally. Therefore, the L:M30 residue was analyzed using mass spectrometry (MS) following storage at 40 °C, for 1 month, of the respective antibody. According to MS, only 2.9% of L:M30 were oxidized, suggesting that L:M30 exhibits only low susceptibility to oxidation. The second mutation, L:P80S, present in C0090 antibody showed a modest increase in surface hydrophobicity (Figure 5C). Notably, this increase was also observed for variants C0075, C0080, C0085 from the first screen, which contained this mutation (Figure 4E).

Based on the summarized data from mAb specificity and affinity to oxMIF, SEC, HIC, AC-SINS analyses, and in silico immunogenicity, C0083 was identified as the most promising mAb variant. It retained the binding properties of imalumab, while exhibiting significantly lower hydrophobicity and aggregation potential and reduced risk of immunogenicity due to the removal of the predicted T-cell epitope 2.

### 3.9. Improvement of Pharmacokinetic (PK) and Biodistribution (BD) Profile of C0083 upon Sequence Optimization

Recently, we evaluated the plasma exposure and in vivo half-life of C0083 in a head-to-head comparison with C0008 in Balb/c nude mice and assessed their tumor penetration and retention in Balb/c mice carrying subcutaneous CT26 tumors [14]. Our findings revealed that C0083 has a 1.4-fold longer terminal half-life (54.1 h) and a 2.3-fold higher plasma exposure (4113 µg/mL*h) compared to imalumab (half-life: 37.4 h; plasma exposure: 1760 µg/mL*h). Additionally, C0083 demonstrated a 1.3-fold extended tumor half-life of 113 h and a 1.7-fold increased tumor penetration of 222 RFU/mm^2^*h, relative to imalumab (tumor half-life: 84 h; tumor penetration: 127 RFU/mm^2^*h). Thus, our engineering efforts significantly improved the PK and BD profiles of C0083, an advancement directly linked to the reduced hydrophobicity and aggregation propensity achieved through targeted mutations in its variable regions.

### 3.10. Analysis of C0083 Crystal Structure to Evaluate the Effects of Selected Mutations

**Figure 6:**
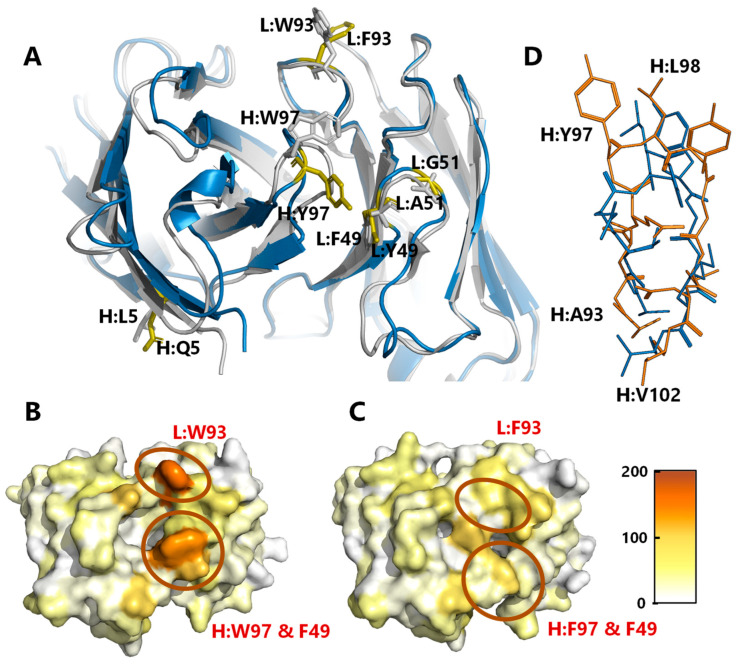
**Crystal structure of C0083.** (**A**) Comparison of the crystal structure of the Fv regions of imalumab (PDB: 6FOE, gray) and C0083 (PDB: 9FQO, blue, mutated amino acids in yellow). Mutated amino acids are shown as sticks and are labeled with the chain ID (H, heavy chain; L, light chain) and numbered according to Kabat. (**B**,**C**) The Fv regions in top view and VH/VL orientation of the crystal structure of imalumab (**B**) and C0083 (**C**) were colored according to the hydrophobicity index (hydrophilic/white–hydrophobic/orange) using the Black and Mould hydrophobicity scale and the solvent accessible area to calculate the hydrophobicity of the individual amino acids. (**D**) Comparison of the H:CDR3 loop of C0083 crystal structure (blue) and C0083 ABodyBuilder model (orange).

To assess the impact of the specified point mutations (LC: F49Y/A51G/W93F; HC: L5Q/W97Y) on the overall structure, the Fab region of C0083 was recombinantly expressed, purified, and crystallized. The X-ray diffraction pattern was collected, and the crystal structure was determined by molecular replacement using the BaxB01 Fab (=imalumab, BAX69; PDB ID: 6FOE). The structure of C0083 was resolved to 1.7 Å (PDB ID: 9FQO, Table S1) and alignment with imalumab revealed that the peptide backbones of both structures were highly similar (Figure 6A). Despite the overall similarity, the H:CDR3 of C0083 was completely restructured due to the mutation H:W97Y, while the L:CDR3 containing the mutation L:W93F did not differ substantially from imalumab. However, the L:W93F mutation in L:CDR3 facilitates an interaction between L:F93 and L:I2, which are now approximately 3 Å apart. This interaction led to a significant decrease in the solvent-accessible surface area (SASA) of L:F93 (C0083, L:F93: 70.9 Å^2^ vs. imalumab, L:W93: 157.0 Å^2^), resulting in reduced exposure of this hydrophobic amino acid. Notably, in the parental antibody, this interaction may be unfavorable for L:W93 due to the presence of its polar NH group in the indole structure. In the completely restructured H:CDR3, H:Y97 adopted a new conformation and occupied the space originally taken by H:Y99 in the parent Fab (Figure 6A). The altered loop conformation enabled an interaction between H:Y97 and L:Y49, and significantly shifted the position of H:Y99. In imalumab, H:Y99 was prominently exposed on the surface, whereas in C0083, it is now buried near L:T94, resulting in a significant reduction in the calculated SASA of H:Y99 in C0083 (21.4 Å^2^) compared to imalumab (110.4 Å^2^) (Figure 6B,C). Furthermore, comparison of the crystal structures of C0083 Fab (9FQO) and the parental imalumab Fab (6FOE) using normalized B-factors reveals that the L:A51G mutation has increased the flexibility of the CDR2 loop (Figure S3). This increased flexibility in C0083 could facilitate the interaction between L:F49 and H:W97, which does not occur in the parental Fab. The interaction of these amino acids in turn could substantially contribute to the observed restructuring of the H:CDR3 loop.

We also compared the solved crystal structure of C0083 to its model Fv-M16 generated by ABodyBuilder (Table 1). While the algorithm successfully modeled most of the Fv region, accurately predicting the conformation and amino acid positions of the H:CDR3 loop proved challenging. As shown in Figure 6D, the positions of amino acids H:Y97, H:L98, and H:Y99 in the C0083 model (Fv-M16) were incorrectly modeled compared to the crystal structure of C0083. This discrepancy resulted in an overestimation of the hydrophobicity score of the H:CDR3 loop (model Fv-M16: 391.8 vs. C0083 crystal structure: 225.9). Additionally, the model slightly overestimated the solvent exposure and thus the hydrophobicity scores of residues H:I31 (model Fv-M16: 119.1 vs. C0083 crystal structure: 84.2) and H:Y32 (model Fv-M16: 61.22 vs. C0083 crystal structure: 22.37) in the H:CDR1, leading to an increased hydrophobicity score (+86.8) when comparing H:CDR1 of model Fv-M16 to the crystal structure of C0083. In summary, relying solely on the models without conducting biophysicochemical in vitro experiments would have precluded C0083 from being selected as a promising candidate.

## 4. Discussion

Macrophage migration inhibitory factor (MIF) has been described as a pleiotropic proinflammatory and protumorigenic cytokine implicated in the pathogenesis of cancer and inflammatory diseases [12]. Due to its ubiquitous presence in the circulation and tissue of healthy subjects and its contribution to many cellular processes [57,58], MIF can be regarded as a challenging target for therapeutic interventions. However, in an oxidative proinflammatory milieu, MIF was shown to convert to oxidized MIF (oxMIF) by posttranslational modification. Oxidation exposes new epitopes creating an immunologically distinct structural isoform known as oxMIF. This isoform is found in patients with inflammatory diseases and solid tumors, making it the disease-related isoform specifically targetable by mAbs [12,13,14,15,17,34,59].

The first generation anti-oxMIF antibody imalumab (synonyms: BAX69, BaxB01) demonstrated promising results in a phase I clinical trial with an acceptable safety profile [19]. However, imalumab showed an unusually short half-life in patients, increased aggregation propensity, and an unfavorable pharmacokinetic profile [19]. A fast clearance, leading to a short half-life, can substantially influence the efficacy and dosage regimen of a therapeutic antibody [60].

Thus, in this study we aimed to identify the molecular characteristics contributing to the reduced half-life of imalumab and to develop an improved second-generation anti-oxMIF antibody through protein engineering. Our strategy for creating an optimized oxMIF-targeting mAb involved combining in silico tools with biophysicochemical in vitro characterization. Specifically, we analyzed the sequences of imalumab’s VH and VL regions using structure- and sequence-based methods to identify amino acids contributing to potential liabilities. The in silico screening identified several amino acids associated with high surface hydrophobicity (H:L5, H:L11, H:I31, H:P41, H:Y58, H:W97, L:V15, L:M30, L:P40, L:F49, L:P80, and L:W93, Figure 1), susceptibility to oxidation (H:W97, L:M30, and L:W93), two potential T-cell epitopes (I_29_MTYLNWYQ_37_ and F_49_VASHSQSG_57_), and residues uncommon in the closest germlines (H:P41, H:S49, H:R83, H:A84, L:D1, L:Q3, L:L11, L:V15, L:P80, and L:S83) (Figure 2).

To address these sequence liabilities, point mutations (Table 1) were introduced into the variable regions of imalumab. Molecular dynamics (MD) simulations were employed to compare the structure of the imalumab Fab with structural prediction models that incorporated the proposed mutations. Model Fv-M1 containing all of these mutations, as well as models with selected combinations (Table 1), showed minimal structural variation in residue positions compared to the parental antibody. However, reduced hydrophobicity scores were observed for specific amino acids in both the heavy chain (HC, L5Q, P41S, and W97Y) and light chain (LC, V15T, F49Y, and W93F). Interestingly, the total hydrophobicity score remained unchanged for the HC and was even increased for the LC (Figure 3). Therefore, experimental characterization was crucial and revealed that W97Y in H:CDR3 and W93F in L:CDR3 led to the most significant decrease in surface hydrophobicity, as demonstrated by size-exclusion chromatography (SEC) and hydrophobic interaction chromatography (HIC) (Figure 4). This reduction in surface hydrophobicity resulted in decreased self-interaction, as shown by affinity-capture self-interaction nanoparticle spectroscopy (AC-SINS). Notably, variants containing both CDR3 mutations—H:W97Y and L:W93F—showed an almost complete elimination of self-interaction. In contrast, variants with just one of these mutations showed only minor reduction. Interestingly, mutations H:W97Y and L:W93F in the CDR3 regions of the respective chains did not affect binding to oxMIF. Conversely, the mutation L:Y36F, designed to remove one of the predicted high affinity T-cell epitopes and located at the VH/VL domain interface at the start of FR2, resulted in a decreased binding to oxMIF and reduced mAb stability.

The variable regions of imalumab were initially identified from a diverse panel of 145 unique MIF-specific antibodies, sourced from the Dyax Fab310 phage display human Fab library [34]. Phage display libraries sometimes encounter suboptimal frameworks. Thus, we introduced mutations in the framework region at residues that were uncommon at their positions relative to the closest germlines or had high hydrophobicity scores. Interestingly, imalumab appears to have relatively optimal frameworks that do not require extensive modifications, as these additional mutations in the framework region (variants C0209-C0212, C0214, and C0216) did not significantly improve mAb characteristics compared to C0083 (Figure 5).

We therefore identified C0083 as the most promising candidate. With only minimal modifications compared to imalumab, it maintained specificity and affinity for oxMIF in the low nanomolar range while exhibiting significantly reduced hydrophobicity and aggregation potential due to the H:L5Q/W97Y and L:F49Y/W93F mutations. Additionally, the L:A51G mutation reduced immunogenicity by eliminating a high-affinity T-cell epitope. The significant improvement in hydrophobicity with the H:W97Y and L:W93F mutations was surprising, given that both the wild-type residues (W) and their substitutions (Y, F) are generally considered hydrophobic. To understand the structural basis for this change, we conducted a crystallization study of the C0083 Fab, which revealed a notable restructuring of the H:CDR3 loop (Figure 6). This restructuring reduced hydrophobicity and aggregation propensity. Despite these structural changes, C0083 maintained its specificity and high affinity for oxMIF.

Antibody variable regions can impact mAb pharmacokinetics, with low solubility due to surface hydrophobicity potentially leading to poor biodistribution and undesirable pharmacokinetic profiles. We have previously reported a head-to-head comparison between C0008, which has the same sequence as imalumab but lacks the C-terminal lysine, and C0083. As delineated in Rossmueller et al. [14], C0083 demonstrated a 1.4-fold longer plasma half-life compared to C0008 in Balb/c nude mice. In line with improved pharmacokinetics in wild-type mice, tumor penetration and retention of C0083 were significantly improved over C0008 in Balb/c mice bearing subcutaneous syngeneic CT26 colon tumors. Thus, removing the exposed hydrophobic patches that drive imalumab’s self-interaction and aggregation significantly extended its half-life and improved its pharmacokinetics in vivo [14]. The variable regions of C0083 were recently incorporated into novel anti-oxMIF mAbs: ON203 [14] and ON105 [61] for cancer treatment and ON104 [13] for treatment of chronic inflammation. These studies revealed that our bioengineering efforts translated into enhanced tumor growth inhibition in prostate cancer mouse models (ON203) [14], strong anticancer activity in pancreatic and colon cancer mouse models using ON105 and applying OncoOne’s pretargeted radioimmunotherapy platform PreTarg-it^®^ [61], and attenuation of clinical and histopathological signs of collagen-induced arthritis (CIA) in a mouse model (ON104) [13]. Further efforts are warranted to validate these mAb candidates in clinical trials.

## 5. Conclusions

By combining in silico analysis with experimental validation, we identified surface hydrophobicity and aggregation propensity as the underlying causes of the unusually short half-life of the first generation anti-oxMIF antibody imalumab. The introduction of point mutations H:L5Q/W97Y and L:F49Y/A51G/W93F into the variable regions of imalumab resulted in substantial improvements in the biophysicochemical properties of the antibody candidates, while maintaining high affinity and specificity for oxMIF. C0083, containing these mutations, was identified as the lead candidate due to its markedly reduced surface hydrophobicity and aggregation potential. Furthermore, it exhibited a minimized risk of immunogenicity and an improved pharmacokinetic profile. The structural analysis of C0083 Fab employing X-ray crystallography demonstrated that the reorganization of the heavy chain CDR3 loop was a crucial factor in the observed enhancement of biophysicochemical characteristics. This work demonstrates how addressing molecular liabilities through bioengineering can optimize antibodies, establishing C0083 and its derivatives as promising candidates for targeted therapies in cancer and inflammatory diseases on the path to clinical validation.

## Figures and Tables

**Table 1:** Overview of the screened anti-oxMIF antibody variants highlighting their mutations compared to C0008 (=imalumab, BaxB01 (PDB ID: 6FOE) and BAX69, GenBank JB325049.1 (LC), GenBank JB325055.1 (HC)). Positions of the VH and VL mutations are numbered according to Kabat.

ID	VL Mutations	VH Mutations	Model ID
C0008	wt	wt	
-	D1A/Q3R/L11F/V15T/M30L/Y36F/F49Y/A51G/P80S/S83F/W93F	L5Q/L11A/G16R/P41S/S49G/R83K/A84T/W97Y/	Fv-M1
**1** **st screen**			
C0069	wt	L5Q	Fv-M2
C0070	wt	L5Q/W97Y	Fv-M3
C0071	F49Y/A51G	wt	Fv-M4
C0072	F49Y/A51G/Y36F	wt	Fv-M5
C0073	F49Y/A51G/W93F	wt	Fv-M6
C0074	F49Y/A51G/Y36F/W93F/M30L	wt	Fv-M7
C0075	F49Y/A51G/Y36F/W93F/M30L/P80S	wt	Fv-M8
C0076	F49Y/A51G	L5Q	Fv-M9
C0077	F49Y/A51G/Y36F	L5Q	Fv-M10
C0078	F49Y/A51G/W93F	L5Q	Fv-M11
C0079	F49Y/A51G/Y36F/W93F/M30L	L5Q	Fv-M12
C0080	F49Y/A51G/Y36F/W93F/M30L/P80S	L5Q	Fv-M13
C0081	F49Y/A51G	L5Q/W97Y	Fv-M14
C0082	F49Y/A51G/Y36F	L5Q/W97Y	Fv-M15
C0083	F49Y/A51G/W93F	L5Q/W97Y	Fv-M16
C0084	F49Y/A51G/Y36F/W93F/M30L	L5Q/W97Y	Fv-M17
C0085	F49Y/A51G/Y36F/W93F/M30L/P80S	L5Q/W97Y	Fv-M18
**2** **nd screen**			
C0090	F49Y/A51G/W93F/M30L/P80S	L5Q/W97Y	Fv-M19
C0209	W93F/L11F/V15T/	W97Y/ L11A	Fv-M20
C0210	W93F	W97Y/ G16R/S49A	Fv-M21
C0211	F49Y/A51G/W93F/ D1A/Q3R/L11F/V15T	L5Q/W97Y/ S49G/P41S/R83K/A84T	Fv-M22
C0212	W93F/S83F	W97Y	Fv-M23
C0213	W93F	W97Y	Fv-M24
C0214	F49Y/A51G/W93F	L5Q/W97Y/ S49G/P41S/R83K/A84T	Fv-M25
C0216	F49Y/A51G/W93F/ D1A/Q3R/L11F/V15T	L5Q/W97Y	Fv-M26

## Data Availability

Data generated in this study are supplied within this manuscript and supplementary data files. Raw data are available on request from the corresponding author. Structure data were deposited to wwPDB (ID: 9FQO).

## Supplementary Materials

The following supporting information can be downloaded at https://www.mdpi.com/article/10.3390/antib13040104/s1: Figure S1: DSF of the antibody variants; Figure S2: SDS-PAGE with Coomassie staining of the antibody variants; Figure S3: b-factor normalization of C0083 (left) and imalumab crystal structure (right); Table S1: Parameters of the C0083 crystallization experiment.
