# Supplementary material for: Integrating In Silico and In Vitro Tools for Optimized Antibody Development—Design of Therapeutic Anti-oxMIF Antibodies
Source: Antibodies (Basel). 2024 Dec 20;13(4):104. doi: 10.3390/antib13040104 (PMC11672567; doi:10.3390/antib13040104)
Supplement: Supplementary file 1 [file antibodies-13-00104-s001.zip › antibodies-3339488-supplementary.pdf]

Supplementary Information

Integrating In Silico and In Vitro Tools for Optimized Antibody Development—Design of Therapeutic Anti-oxMIF Antibodies

Gregor Rossmueller[1], Irina Mirkina[1], Michael Thiele[1], Alejandro Puchol Tarazona[1], Florian Rueker[2], Randolf J. Kerschbaumer[1], Alexander Schinagl[1*]

[1]OncoOne Research & Development GmbH, Karl-Farkas-Gasse 22, A-1030 Vienna, Austria.

[2]University of Natural Resources and Life Sciences, Vienna, Department of Biotechnology, Institute of Molecular Biotechnology, Muthgasse 18, A-1190 Vienna, Austria

***Corresponding Author:** Alexander Schinagl, PhD, OncoOne Research & Development GmbH, Karl-Farkas-Gasse 22, A-1030 Vienna, Austria, Phone: +43 1 9092208-6000; E-mail: alexander.schinagl@oncoone.com

DSF analysis

A mixture containing 5 µL of Sypro Orange (diluted at 1/200 in water; Sigma), 5 µL of antibodies at a concentration of 0.1 mg/mL, and 40 µL of 250 mM glycine buffer at pH 5.0, was prepared in a tube. The tubes were securely sealed with an optical flat cap and heated using an i-Cyler iQ5 real-time PCR detection system (Bio-Rad), from 20 to 95°C in 1°C increments. Fluorescence changes in the plate wells were monitored with a charge-coupled camera. The excitation and emission wavelengths were set at 485 nm and 575 nm, respectively. The temperature midpoint (T_m) for the protein unfolding transition was determined using the Bio-Rad iQ5 software.

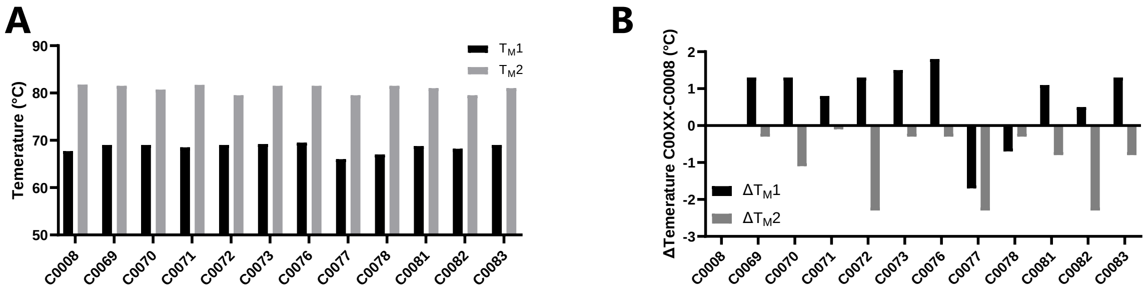

Figure S1: DSF of the antibody variants. (A) The unfolding temperatures (TM) of the antibody variants for the TM1 in black corresponds to the CH2 unfolding and TM2 in grey to the CH3/Fab unfolding. **(B)** The difference between C0008 and the antibody variants for their unfolding temperatures.

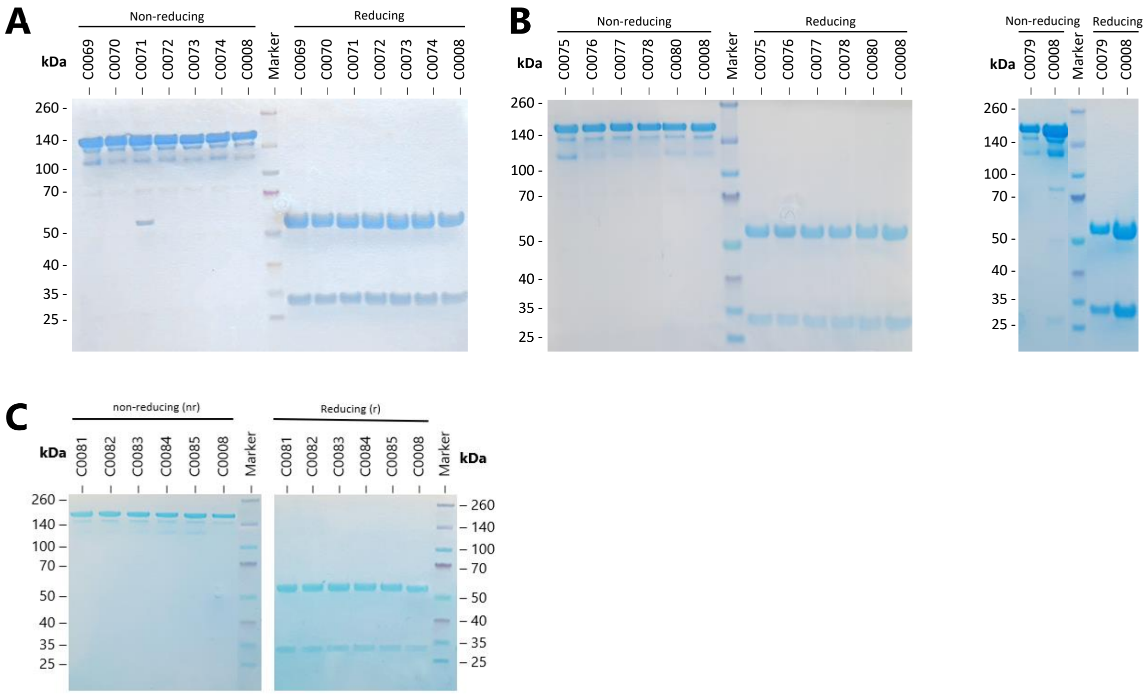

Figure S2: SDS-PAGE with Coomassie staining of the antibody variants. (A) Variants C0069-C0074 and C0008. **(B)** Variants C0075-C0080 and C0008. **(C)** C0081-C0085 and imalumab.

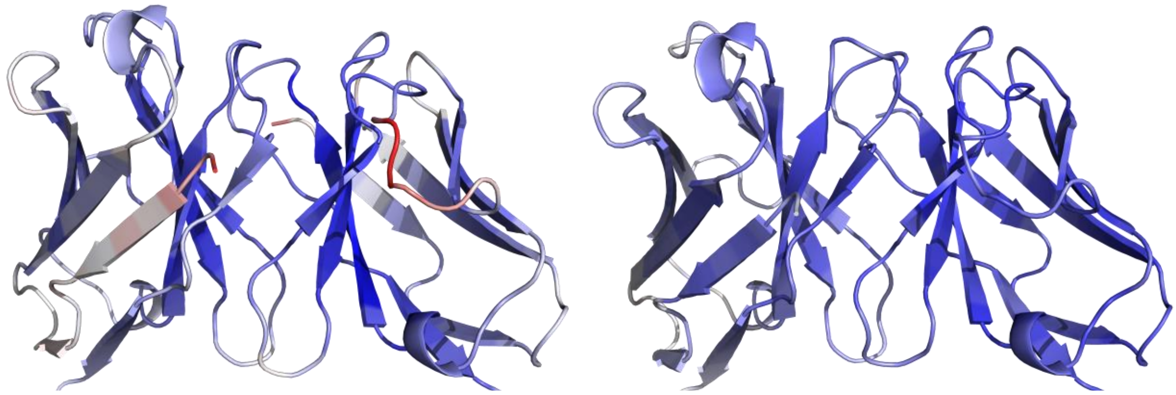

Figure S3: b-factor normalization of C0083 (left) and imalumab crystal structure (right). The normalized B-factors are shown as a color spectrum (blue-white-red) from -2 to 4. The B-factors are measures to describe the attenuation of X-ray scattering due to the thermal motion of atoms. Increased signal attenuation correlates with enhanced atomic motion, indicating a greater likelihood of these atoms residing in more flexible regions of the overall structure.

Supplementary Table S1: Parameters of the C0083 crystallization experiment.

	**C0083-Fab**
**Wavelength (nm)**	0.08731
**Resolution range**	41.93 - 1.7 (1.761 - 1.7)
**Space group**	P 1 21 1
Unit cell<br>a, b, c (Å)<br>α, β, γ (°)	 51.54 66.36 58.65<br>90 100.1 90
**Total reflections**	289442 (29483)
**Unique reflections**	42855 (4260)
**Multiplicity**	6.8 (6.9)
**Completeness (%)**	99.72 (99.51)
**Mean I/sigma(I)**	9.22 (1.44)
**Wilson B-factor**	27.64
**R-merge**	0.175 (1.647)
**R-meas**	0.19 (1.781)
**R-pim**	0.07316 (0.6726)
**CC1/2**	0.995 (0.705)
**CC***	0.999 (0.909)
**Reflections used in refinement**	42790 (4247)
**Reflections used for R-free**	929 (92)
**R-work**	0.2154 (0.3756)
**R-free**	0.2367 (0.3668)
**CC(work)**	0.957 (0.813)
**CC(free)**	0.926 (0.766)
**Number of non-hydrogen atoms**	3615
**macromolecules**	3341
**ligands**	33
**solvent**	260
**Protein residues**	436
**RMS(bonds)**	0.002
**RMS(angles)**	0.51
**Ramachandran favored (%)**	96.3
**Ramachandran allowed (%)**	3.7
**Ramachandran outliers (%)**	0
**Rotamer outliers (%)**	0.52
**Clashscore**	1.36
**Average B-factor**	37.6
**macromolecules**	37.31
**ligands**	51.49
**solvent**	40.6
**Number of TLS groups**	2
